# Antioxidative phytochemicals from *Rhododendron oldhamii* Maxim. leaf extracts reduce serum uric acid levels in potassium oxonate-induced hyperuricemic mice

**DOI:** 10.1186/s12906-015-0950-7

**Published:** 2015-12-01

**Authors:** Yu-Tang Tung, Lei-Chen Lin, Ya-Ling Liu, Shang-Tse Ho, Chi-Yang Lin, Hsiao-Li Chuang, Chien-Chao Chiu, Chi-Chang Huang, Jyh-Horng Wu

**Affiliations:** Graduate Institute of Sports Science, National Taiwan Sport University, Taoyuan, 333 Taiwan; Department of Forestry and Natural Resources, National Chiayi University, Chiayi, 600 Taiwan; Department of Forestry, National Chung Hsing University, Taichung, 402 Taiwan; National Laboratory Animal Center, National Applied Research Laboratories, Taipei, 11529 Taiwan; Division of Animal Resource, Animal Technology Laboratories, Agricultural Technology Research Institute, Hsinchu City, 30093 Taiwan

**Keywords:** *Rhododendron oldhamii* maxim, Leaf, Phytochemical, Online HPLC–DPPH, Hyperuricemia

## Abstract

**Background:**

Some of the genus *Rhododendron* was used in traditional medicine for arthritis, acute and chronic bronchitis, asthma, pain, inflammation, rheumatism, hypertension and metabolic diseases and many species of the genus *Rhododendron* contain a large number of phenolic compounds and antioxidant properties that could be developed into pharmaceutical products.

**Methods:**

In this study, the antioxidative phytochemicals of *Rhododendron oldhamii* Maxim. leaves were detected by an online HPLC–DPPH method. In addition, the anti-hyperuricemic effect of the active phytochemicals from *R. oldhamii* leaf extracts was investigated using potassium oxonate (PO)-induced acute hyperuricemia.

**Results:**

Six phytochemicals, including (2*R*, 3*R*)-epicatechin (**1**), (2*R*, 3*R*)-taxifolin (**2**), (2*R*, 3*R*)-astilbin (**3**), hyposide (**4**), guaijaverin (**5**), and quercitrin (**6**), were isolated using the developed screening method. Of these, compounds **3**, **4**, **5**, and **6** were found to be major bioactive phytochemicals, and their contents were determined to be 130.8 ± 10.9, 105.5 ± 8.5, 104.1 ± 4.7, and 108.6 ± 4.0 mg per gram of EtOAc fraction, respectively. In addition, the four major bioactive phytochemicals at the same dosage (100 mmol/kg) were administered to the abdominal cavity of potassium oxonate (PO)-induced hyperuricemic mice, and the serum uric acid level was measured after 3 h of administration. H&E staining showed that PO-induced kidney injury caused renal tubular epithelium nuclear condensation in the cortex areas or the appearance of numerous hyaline casts in the medulla areas; treatment with 100 mmol/kg of EtOAc fraction, (2*R*, 3*R*)-astilbin, hyposide, guaijaverin, and quercitrin significantly reduced kidney injury. In addition, the serum uric acid level was significantly suppressed by 54.1, 35.1, 56.3, 56.3, and 53.2 %, respectively, by the administrations of 100 mmol/kg EtOAc fraction and the derived major phytochemicals, (2*R*, 3*R*)-astilbin, hyposide, guaijaverin, and quercitrin, compared to the PO group. The administration of 10 mg/kg benzbromarone, a well-known uricosuric agent, significantly reduced the serum uric acid level by 45.5 % compared to the PO group.

**Conclusion:**

The in vivo decrease in uric acid was consistent with free radical scavenging activity, indicating that the major phytochemicals of *R. oldhamii* leave extracts and the derived phytochemicals possess potent hypouricemic effects, and they could be potential candidates for new hypouricemic agents.

## Background

The major function of xanthine oxidase (XOD) is to catalyze the oxidation of hypoxanthine to xanthine and to further catalyze the oxidation of xanthine to uric acid in purine metabolism [1]. Excess amounts of uric acid in the body result in the deposition of urate crystals in the joints and kidneys, causing inflammation as well as gouty arthritis and uric acid nephrolithiasis [2, 3]. Recent studies also noted that hyperuricemia is associated with a risk of chronic nephritis, renal dysfunction, cardiovascular diseases, hypertension, diabetes, and metabolic syndrome [4, 5]. Thus, there has been increasing interest in the search of more effective or novel bioactive compounds in order to improve the insufficient uric acid excretion from a wide variety of traditional herbal plants [6–8].

The genus *Rhododendron* is widely distributed throughout most of the world, with the exception of Africa and South America [9]. Some members of the genus *Rhododendron* were used in traditional medicine for arthritis, acute and chronic bronchitis, asthma, pain, inflammation, rheumatism, hypertension, and metabolic diseases [10–12]. In addition, *Rhododendron sp.* used in Unani system of medicine for treatment of gout [13, 14]. Recent studies have shown that many species of the genus *Rhododendron* contain a large number of phenolic compounds and antioxidant properties that could be developed into pharmaceutical products [15, 16]. In addition, recent studies noted that phenolics are strong XOD-inhibitors [17] and have the potential to lower the risk of hyperuricemia and gout [18]. In previous studies, it was found that *Rhododendron yedoense* contains large amounts of flavonoids and shows excellent XOD-inhibitory activities [19]. Therefore, methanolic extracts of *R. oldhamii* leaves may be good candidates for further development as clinically relevant anti-hyperuricemic agents. However, to the best of our knowledge, there are no prior reports on *R. oldhamii* leaf extracts and its derivatives possessing antioxidant activity in vitro and hyperuricemic activity in vivo. Therefore, in this study, we investigated the hypouricemic effect of a methanolic extract and its major phytochemicals from *R. oldhamii* leaves in mice for the first time.

## Methods

### Chemicals

Benzbromarone, potassium oxonate, 1,1’-diphenyl-2-picrylhydrazyl radical (DPPH^.^), hypoxanthine, xanthine oxidase, nitroblue tetrazolium chloride (NBT), Folin-Ciocalteu reagent, potassium dihydrogen phosphate (KH_2_PO_4_), and (+)-catechin were purchased from Sigma Chemical Co. (St. Louis, MO, USA). The other chemicals and solvents used in this experiment were HPLC-grade.

### Plant materials

The leaves of *Rhododendron oldhamii* Maxim. from Lion Head Mountain of Taipei county in Taiwan (Lat. 24°56'16"N., Long. 121°30'07"E.) were collected at the end of April 2011. The voucher specimen (voucher no. 6) was deposited at the herbarium of the Department of Forestry and Natural Resources, National Chiayi University (NCYU), Taiwan. The species were identified by Dr. Lei-Chen Lin (NCYU). The materials were air dried at ambient temperature (25 °C) and stored in a refrigerator at 4 °C prior to treatments.

### Extraction, fractionation, and isolation

Leaves were soaked in methanol at ambient temperature for 7 days. The extracts were decanted and filtered through Whatman No.1 filter paper, and the filtrates were concentrated in a rotary evaporator and then lyophilized. Furthermore, the resulting methanolic crude extracts of *R. oldhamii* were successively fractionated with *n*-hexane, ethyl acetate (EtOAc), *n*-butanol (BuOH), and water to yield soluble fractions of hexane, EtOAc, BuOH, and water. The phytochemicals from the EtOAc fraction were separated and purified by semi-preparative HPLC on a Jasco PU-2080 instrument (Tokyo, Japan) equipped with a MD-2010 photo-diode array detector (Jasco) and a 250 mm × 10.0 mm i.d., 5-μm Supelco RP-amide column (Bellefonte, PA, USA). The mobile phase was solvent **A**, 100 % MeOH; and solvent **B**, ultrapure water. The elution conditions were 0–15 min of 30–60 % **A** to **B** (linear gradient); 15–20 min of 60–65 % **A** to **B** (linear gradient); 20–27 min of 65–100 % **A** to **B** (linear gradient); and 27–40 min of 100–100 % **A** to **B** at a flow rate of 4 mL/min. ESI-MS data were collected by a Finnigan MAT-95S mass spectrometer, and NMR spectra were recorded by a Bruker Avance 500 MHz FT-NMR spectrometer. The structures of compound **1**, compound **2**, compound **3**, compound **4**, compound **5**, and compound **6** were identified by ESI-MS and NMR.

### Quantification

The phytochemicals were quantified by LC-MS (Thermo, Dioxed UltiMate 3000 UHPLC; Bruker, amazon speed) with a 250 mm × 4.6 mm i.d., 2.6-μm C-18 column (Phenomenex, Torrance, CA, USA). The mobile phase was solvent **A**, 100 % MeOH; and solvent **B**, ultrapure water. The elution conditions were 0–15 min of 30–60 % **A** to **B** (linear gradient); 15–20 min of 60–65 % **A** to **B** (linear gradient); 20–27 min of 65–100 % **A** to **B** (linear gradient); and 27–40 min of 100–100 % **A** to **B** at a flow rate of 0.5 mL/min using a detector. For the preparation of the calibration curve, standard stock solutions of compounds were prepared in methanol, filtered through Millipore filters (0.45 μm), and appropriately diluted to obtain the desired concentrations in the quantification range. The calibration graphs were plotted after the linear regression of the peak areas versus concentrations.

### DPPH radical-scavenging activity (DPPH assay)

The scavenging activity of the DPPH free radical by the test samples was determined according to the method reported by Ho et al. [20]. Ten μL of the test samples was mixed with 200 μL of 0.1 mM DPPH-ethanol solution and 90 μL of 50 mM Tris–HCl buffer (pH 7.4). Methanol (10 μL) alone was used as the control in this experiment. After 30 min of incubation at room temperature, the reduction in DPPH free radicals was measured by reading the absorbance at 517 nm using a Thermo Scientific Multiskan GO microplate reader (Vantaa, Finland). (+)-Catechin was used as the positive control. The inhibition ratio was calculated using the following equation: % inhibition = [(absorbance of control – absorbance of test sample)/ absorbance of control] × 100.

### Superoxide radical-scavenging activity (NBT assay)

The measurement of superoxide radical-scavenging activity was carried out using the method described by Tung et al. [21], and (+)-catechin was used as the standard. First, 20 μL of 15 mM Na_2_EDTA in buffer (50 mM KH_2_PO_4_/KOH, pH 7.4), 50 μL of 0.6 mM NBT in buffer, 30 μL of 3 mM hypoxanthine in 50 mM KOH, 5 μL of the test samples in methanol, and 145 μL of buffer were mixed in 96-well microplates (Falcon, USA). The reaction was initiated by adding 50 μL of a xanthine oxidase solution in buffer (1 unit in 10 mL buffer) to the mixture. The reaction mixture was incubated at room temperature, and the absorbance at 570 nm was determined every 20 s up to 5 min using a plate reader. The control was 5 μL of methanol instead of the sample solution. (+)-Catechin was used as the positive control. The inhibition ratio was calculated using the following equation: %  Inhibition = [(rate of control – rate of sample reaction)/ rate of control] × 100.

### Reducing power assay

This assay was determined according to the method reported by Lin et al. [16], with slight modifications. Briefly, 1 mL of the reaction mixture containing 500 μL the test extracts or compounds in 500 μL phosphate buffer (0.2 M, pH 6.6) was incubated with 500 μL potassium ferricyanide (1 %, w/v) at 50 °C for 20 min. The reaction was terminated by adding trichloroacetic acid (10 %, w/v), and the mixture was centrifuged at 3000 rpm for 10 min. The supernatant solution (500 μL) was mixed with distilled water (500 μL) and 100 μL of ferric chloride (0.1 %, w/v) solution, and the absorbance was measured at 700 nm. The reducing ability was expressed as (+)-catechin equivalents (CE) in milligrams per gram sample.

### Determination of total phenolics

The total phenolic content was determined according to the Folin-Ciocalteu method [16], using gallic acid as the standard. The test sample (5 mg) was dissolved in 5 mL of methanol/water (50:50, v/v). The extract solution (500 μL) was mixed with 500 μL of 50 % Folin-Ciocalteu reagent. The mixture was kept for a 5-min period, which was followed by the addition of 1.0 mL of 20 % Na_2_CO_3_. After 10 min of incubation at room temperature, the mixture was centrifuged for 8 min (1200 *g*) and the absorbance of the supernatant was measured at 730 nm. The total phenolic content was expressed as gallic acid equivalents (GAE) in milligrams per gram sample.

### On-line DPPH radical-scavenging analysis

The best antioxidant activity of the extract (EA fraction) from *R. oldhamii* leaves was further monitored by the on-line RP–HPLC–DPPH method. The EA fraction (stock concentration = 20 mg/mL) was monitored by analytic HPLC on a model PU-2080 instrument (Jasco, Japan) with a 250 mm × 10.0 mm i.d., 5-μm Supelco RP-amide column (Bellefonte, PA, USA). The mobile phase was solvent **A**, 100 % MeOH; and solvent **B**, ultrapure water. The elution conditions were 0–15 min of 30–60 % **A** to **B** (linear gradient); 15–20 min of 60–65 % **A** to **B** (linear gradient); 20–27 min of 65–100 % **A** to **B** (linear gradient); and 27–40 min of 100–100 % **A** to **B** at a flow rate of 4 mL/min, using a Jasco MD-2010 photo diode array at 280 nm wavelength. For the on-line DPPH radical-scavenging analysis, the flow of DPPH reagent (300 mg/L in methanol) was set to 4 mL/min, and the induced bleaching was detected photometrically as a negative peak at 517 nm.

### Animals

Male ICR mice with a body weight of approximately 30 g (8 weeks old) were purchased from BioLASCO (A Charles River Licensee Corp., Yi-Lan, Taiwan). Mice were given a standard laboratory diet and distilled water *ad libitum*, and they were kept on a 12-h light/dark cycle at 22 ± 2 °C. All animal experimental protocols were approved by the Institutional Animal Care and Use Committee (IACUC) of National Taiwan Sport University, and the study conformed to the guidelines of the protocol IACUC-10319 approved by the IACUC ethics committee.

### Potassium oxonate (PO) − induced hyperuricemia in mice

For the hyperuricemia study, potassium oxonate (PO), an uricase inhibitor, was employed to induce acute hyperuricemia according to Tung et al. [22]. Sixty-four mice were randomly assigned to eight groups for treatment (*n* = 8 per group): (1) vehicle group; (2) PO group; (3) PO + BZM group; (4) PO + EA group; (5) PO + AS group; (6) PO + HP group; (7) PO + GJ group; and (8) PO + QR group. Briefly, mice were intraperitoneally (*i.p.*) injected with PBS containing PO (200 mg/kg) 1 h before the test samples were administered to increase the serum uric acid level. For a comparative study, the same dosage at 100 mmol/kg of the EtOAc fraction (EA, 45.0 mg/kg), (2R, 3R)-astilbin (AS, 45.0 mg/kg), hyposide (HP, 46.4 mg/kg), guaijaverin (GJ, 43.4 mg/kg), and quercitrin (QR, 44.8 mg/kg) dissolved in DMSO were delivered *i.p.* for 1 h post PO administration. In this study, 10.0 mg/kg benzbromarone (BEM), a well-known uricosuric agent, was used as the reference control.

### Pathological histology

Kidney tissue was fixed in 10 % buffered formaldehyde and examined using hematoxylin and eosin (H&E) staining.

### Measurement of serum BUN, creatinine, and uric acid level

Blood samples were collected by retro-orbital bleeding after 3 h PO administration. Blood samples were centrifuged at 1,400 × *g* at 4 °C for 15 min, and the levels of BUN, creatinine and uric acid in the serum supernatants were measured using an autoanalyzer (Hitachi 7060, Hitachi, Japan).

### Statistical analysis

The data for in vitro and in vivo assays are expressed as the mean ± SD (*n* = 3) and the mean ± SEM (*n* = 8), respectively. The significance of difference was calculated by Scheffe’s test, and the results with *P* < 0.05 were considered to be statistically significant.

## Results and discussion

### DPPH radical-scavenging activity of *R. oldhamii* leaf extract and its derived soluble fractions

**Fig. 1:**
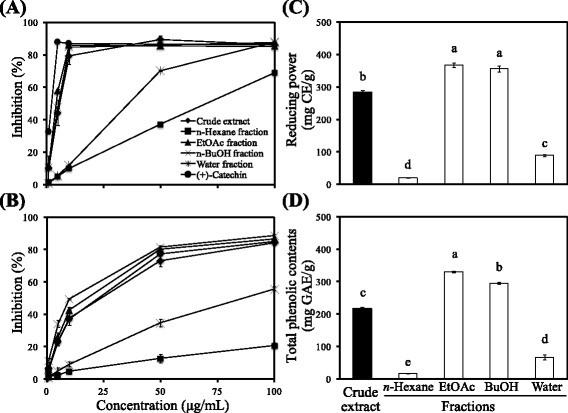
Antioxidant activities of the methanolic extract and its derived soluble fractions from the leaves of *R. oldhamii*. **a** DPPH radical scavenging activity. **b** Superoxide radical scavenging activity. **c** Reducing power. **d** Total phenolic contents. The results are presented as the mean ± SD (*n* = 3). The bars marked by different letters are significantly different at the level of *P* < 0.05, according to Scheffe’s test

In the present study, the free radical-scavenging activity of *R. oldhamii* leaf extract was assessed by a DPPH assay. Accordingly, as shown in Fig. 1a, the DPPH radical-scavenging activity of the methanolic extract and its derived soluble fractions from leaves of *R. oldhamii*, including the soluble fractions of *n*-hexane, EtOAc, BuOH, and water, were shown in a dose-dependent manner. Of these, the EtOAc fraction showed the strongest activity. Meanwhile, except for the *n*-hexane and water-soluble fractions, all extracts showed a good inhibitory activity against the DPPH radicals. The IC_50_ values (the concentration required to inhibit radical formation by 50 %) of the crude extract, hexane, EtOAc, BuOH, and water fraction were 7.5 ± 0.2, 77.6 ± 2.3, 5.0 ± 0.1, 6.1 ± 0.1 and 30.0 ± 0.6 μg/mL, respectively. The IC_50_ value of (+)-catechin, a well-known antioxidant compound used as the reference control in this study, was 1.9 ± 0.0 μg/mL. Lin et al. [16] reported that the IC_50_ values of the crude extracts of *Rhododendron* species increased in the following order: *R. pseudochrysanthum* (7.5 μg/mL), *R. oldhamii* (7.5 μg/mL), *R. kanehirai* (7.7 μg/mL), *R. breviperulatum* (8.8 μg/mL), *R. rubropilosum* var. *taiwanalpinum* (10.4 μg/mL), *R. formosanum* (10.7 μg/mL), *R. simsii* (11.8 μg/mL), *R. rubropilosum* var. *rubropilosum* (12.1 μg/mL), *R. ellipticum* (14.2 μg/mL), and *R. mariesii* (14.7 μg/mL). These results showed that the crude extracts of the *Rhododendron* species exhibited a good DPPH radical scavenging activity.

### Superoxide radical-scavenging activity of *R. oldhamii* leaf extract and its derived soluble fractions

Superoxide radical was generated by the hypoxanthine-xanthine oxidase and NBT systems in this assay. Figure 1b shows the superoxide radical-scavenging activity of the methanolic extract and its derived fractions from *R. oldhamii* leaves compared with (+)-catechin. At the 10 μg/mL test concentration, the superoxide radical inhibition of *R. oldhamii* leaf extract and its derived fractions decreased in the following order: BuOH fraction (49.6 %) > EtOAc fraction (42.6 %) > crude extract (37.7 %) > water fraction (9.1 %) > *n*-hexane fraction (4.7 %). The IC_50_ values of (+)-catechin, crude extract, *n*-hexane fraction, EtOAc fraction, BuOH fraction, and water fraction were 15.1 ± 0.5, 18 ± 2.1, 433.1 ± 9.0, 11.1 ± 0.4, 9.6 ± 0.6, and 60.1 ± 4.8 μg/mL, respectively. The comparisons of these data revealed that the crude extract, the EtOAc fraction, and the BuOH fraction examined in this study exhibited great superoxide radical-scavenging activity. The present study revealed that the EtOAc fraction and the BuOH fraction showed excellent performances in superoxide radical inhibition and their inhibitory activities were better than that of (+)-catechin. Lin et al. [16] showed that among all soluble fractions from *R. pseudochrysanthum* leaves, both the EtOAc fraction and the BuOH fraction exhibited the best superoxide radical-scavenging activity. This may be because the major components of the EtOAc and BuOH fractions were flavonoids and phenolic acids, which have great superoxide radical-scavenging effects.

### Reducing power of *R. oldhamii* leaf extract and its derived soluble fractions

The reducing power of the crude extract and its derived fractions was calculated as (+)-catechin equivalents (CE) in milligrams per gram sample. As shown in Fig. 1c, the reducing power of the EtOAc fraction (367.9 ± 6.3 mg/g) was higher than that of the BuOH fraction (357.4 ± 9.3 mg/g), the crude extract (285.2 ± 4.1 mg/g), the water fraction (89.3 ± 2.8 mg/g), and the hexane fraction (19.5 ± 0.5 %). These results revealed that the EtOAc fraction possessed the highest antioxidant activity, which was the same as the DPPH radical-scavenging activity results. These results imply that there is an abundance of antioxidative phytochemicals present in the EtOAc fraction.

### Total phenolic contents of *R. oldhamii* leaf extract and its derived soluble fractions

Phenolic compounds are commonly found in the plant kingdom, and they have been reported to have multiple biological effects [23, 24]. A correlation between the content of phenolic compounds and antioxidant activities has been described in many studies [25–27]. The phenolic compounds are very important plant constituents because of their ability to scavenge free radicals. Figure 1d shows the content of total phenolics in the crude extract and its derived fractions calculated as gallic acid equivalents (GAE) in milligrams per gram of sample. Apparently, the total phenolic content of the EtOAc fraction (330.3 ± 3.1 mg/g) was higher than that of the BuOH fraction (295.4 ± 2.9 mg/g), the crude extract (217.6 ± 2.4 mg/g), the water fraction (66.4 ± 7.2 mg/g), and the hexane fraction (16.6 ± 0.6 mg/g). These results imply that there were abundant antioxidative phytochemicals present in the leaf extract of *R. oldhamii*, especially in the EtOAc fraction.

### On-line RP–HPLC–DPPH method

**Fig. 2:**
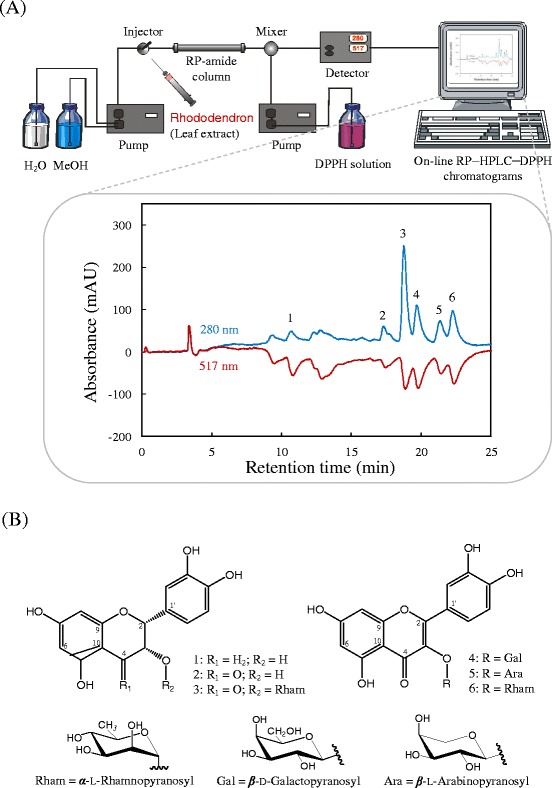
**a** The online HPLC–DPPH chromatograms of the EtOAc fraction from *R. oldhamii* leaf extract. **b** Isolated and identified phytochemicals: (2*R*, 3*R*)-epicatechin (**1**), (2*R*, 3*R*)-taxifolin (**2**), (2*R*, 3*R*)-astilbin (**3**), hyposide (**4**), guaijaverin (**5**), and quercitrin (**6**)

The on-line RP–HPLC–DPPH method can be used for a rapid assessment of pure antioxidant compounds in complex mixtures, particularly plant extracts [28]. The more rapidly the absorbance decreases, the more potent the antioxidant activity of the compound will be in terms of hydrogen-donating ability [29]. The combined UV (positive signals) and DPPH^•^ quenching (negative signals) chromatograms under gradient conditions of the EtOAc fraction from the leaf extract of *R. oldhamii* are presented in Fig. 2a. Several eluted phytocompounds in the EtOAc fraction were detected and gave positive peaks on the UV detector (280 nm). Among them, (2*R*, 3*R*)-epicatechin (**1**), (2*R*, 3*R*)-taxifolin (**2**), (2*R*, 3*R*)-astilbin (**3**), hyposide (**4**), guaijaverin (**5**), and quercitrin (**6**) (Fig. 2b) showed a hydrogen-donating capacity (negative peak) towards the DPPH radical at the applied concentration. The results revealed that the method can be applied for a quick screening of antioxidant compounds or to more precisely determine the radical-scavenging activity of compounds. Thus, it is no longer necessary to isolate and purify non-target phytocompounds, leading to very significant reductions in costs and faster results.

### Quantification and antioxidant activities of major active compounds in leaf extract of *R. oldhamii*

According to the screening results from the on-line RP–HPLC–DPPH system, (2*R*, 3*R*)-epicatechin (**1**), (2*R*, 3*R*)-taxifolin (**2**), (2*R*, 3*R*)-astilbin (**3**), hyposide (**4**), guaijaverin (**5**) and quercitrin (**6**) were found to be the major bioactive phytochemicals in the EtOAc fraction, and their contents were determined to be 15.0 ± 0.3, 27.0 ± 0.4, 130.8 ± 10.9, 105.5 ± 8.5, 104.1 ± 4.7, and 108.6 ± 4.0 mg per gram of EtOAc fraction, respectively (Table 1). To determine the antioxidant activities of these major active compounds, DPPH and NBT assays were performed. As shown in Table 1, the IC_50_ values for the DPPH radical-scavenging activity of these major phytochemicals were 8.4 ± 0.2, 7.6 ± 0.2, 8.2 ± 0.5, 6.8 ± 0.4, 7.4 ± 0.1, and 6.9 ± 0.2 μM, respectively. However, for superoxide radical-scavenging activity, the IC_50_ values of these compounds were 34.1 ± 1.3, 17.9 ± 1.0, 33.2 ± 1.7, 16.1 ± 0.3, 15.8 ± 0.4, and 13.0 ± 0.9 μM, respectively. The DPPH and NBT assays showed that compounds **4**, **5** and **6** had better activities. These results demonstrated that flavonoids containing the 2,3 double bond in the C ring have better antioxidant activities. In contrast, flavans have a saturated heterocyclic C ring, and the consequent lack of conjugation between the A and B rings means that electrons are less able to be delocalized from the B ring to the A ring, thus lowering their antioxidant activities.

**Table 1:** Antioxidant activities and contents of major phytochemicals of the EtOAc fraction from *R. oldhamii* leaves

Phytochemicals	Content (mg/g of EtOAc fraction)	IC_50_ (μM)	
		DPPH radical scavenging	Superoxide radical scavenging
(2*R*, 3*R*)-Epicatechin (**1**)	15.0 ± 0.3	8.4 ± 0.2^a^	34.1 ± 1.3^a^
(2*R*, 3*R*)-Taxifolin (**2**)	27.0 ± 0.4	7.6 ± 0.2^abc^	17.9 ± 1.0^b^
(2*R*, 3*R*)-Astilbin (**3**)	130.8 ± 10.9	8.2 ± 0.5^ab^	33.2 ± 1.7^a^
Hyperoside (**4**)	105.5 ± 8.5	6.8 ± 0.4^c^	16.1 ± 0.3^bc^
Guaijaverin (**5**)	104.1 ± 4.7	7.4 ± 0.1^bc^	15.8 ± 0.4^bc^
Quercitrin (**6**)	108.6 ± 4.0	6.9 ± 0.2^c^	13.0 ± 0.9^c^

Different letters within a column indicate significant difference at the *P* < 0.05 level according to Scheffe’s test

### Anti-hyperuricemic effect in hyperuricemic mice

**Fig. 3:**
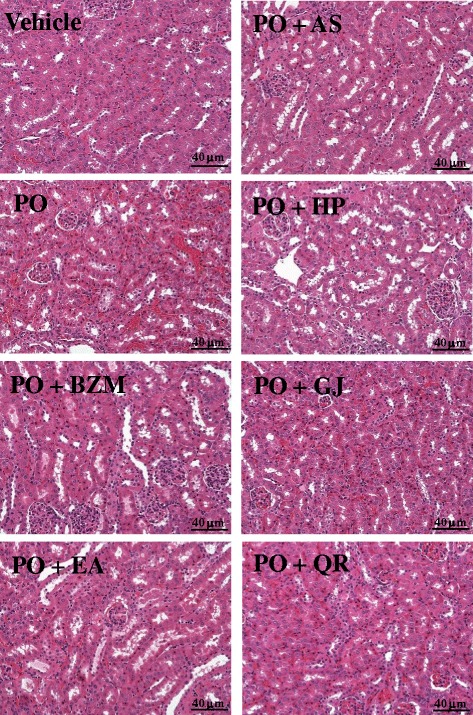
The pathological histology of the EtOAc fraction and its derived phytochemicals of *R. oldhamii* leaf extract in PO-induced hyperuricemic mice. The H&E staining showed that PO-induced kidney injury caused renal tubular epithelial nuclear condensation (karyopyknosis) in the cortex or the appearance of numerous hyaline casts in the medulla areas. The levels of renal tubular epithelial nuclear condensation or numerous hyaline casts were significantly reduced by benzbromarone, the EtOAc fraction, (2*R*, 3*R*)-astilbin, hyposide, guaijaverin, and quercitrin treatments

**Fig. 4:**
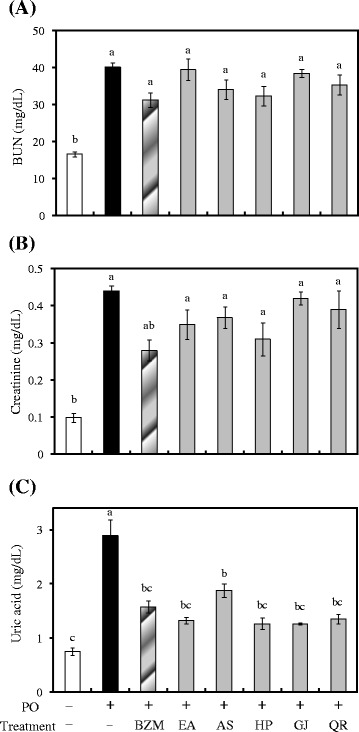
The reductions in serum BUN, creatinine, and the uric acid level by the EtOAc fraction and its derived phytochemicals of *R. oldhamii* leaf extract in PO-induced hyperuricemic mice. The results are presented as the mean ± SEM of eight mice. Different letters are significantly different at the level of *P* < 0.05 according to the Scheffe’s test

Benzbromarone and allopurinol are widely used for the treatment of hyperuricemia. Allopurinol inhibits ROS production by working as an XO inhibitor but not as a radical scavenger, whereas benzbromarone decreases ROS production directly and indirectly by working as both a uric acid transporter 1 inhibitor and radical scavenger [30]. In this study, the major compounds of *R. oldhamii* leaf extract were good radical scavengers. Thus, the anti-hyperuricemic effect of *R. oldhamii* leaf extract and its phytochemicals on PO-induced hyperuricemia in mice was studied, and benzbromarone was the reference control used in this study. The hyperuricemic mice were generated by a single intraperitoneal injection of PO (200 mg/kg). PO, a urate oxidase inhibitor, can raise the serum uric acid concentration by inhibiting the decomposition of uric acid by uricase [31]. H&E staining showed that PO-induced kidney injury caused renal tubular epithelium nuclear condensation (karyopyknosis) in the cortex or the appearance of numerous hyaline casts in the medulla areas (Fig. 3). The levels of renal tubular epithelial nuclear condensation or numerous hyaline casts were significantly reduced after the administration of 100 mmol/kg of EtOAc fraction, (2*R*, 3*R*)-astilbin, hyposide, guaijaverin, and quercitrin. Xiong et al. [32] also exhibited that NF-κB plays a role in the pathogenesis of chronic glomerulonephritis, and *rhododendron* root may attenuate renal damages by downregulating the activation of NF-κB. The hyperuricemic animals 3 h after *i.p.* of PO exhibited higher serum levels of BUN (40.2 ± 1.2 mg/dL), creatinine (0.44 ± 0.02 mg/dL), and uric acid (2.9 ± 0.3 mg/dL) when compared to the vehicle control group (16.6 ± 0.7, 0.10 ± 0.01, and 0.8 ± 0.1 mg/dL, respectively). Therefore, at 3 h post PO injection, the serum uric acid level showed a more than 3-fold increase compared with the vehicle control (*P* < 0.05). The administration of benzbromarone (10 mg/kg) significantly reduced the serum uric acid level by 45.5 % compared to the PO group. At an equimolar dose (100 mmol/kg), animals treated with the EtOAc fraction, (2*R*, 3*R*)-astilbin, hyposide, guaijaverin, and quercitrin showed a significant reduction in uric acid by 54.1, 35.1, 56.3, 56.3, and 53.2 %, respectively, compared to the PO group (*P* < 0.05) (Fig. 4). In addition, treating hyperuricemic mice with benzbromarone, the EtOAc fraction, (2*R*, 3*R*)-astilbin, hyposide, guaijaverin, and quercitrin slightly reduced both creatinine and uric acid serum levels. Wang et al. [33] reported that cinnamaldehyde had good inhibitory activity on xanthine oxidase activity and could significantly reduce the serum uric acid level by 60 % compared with the PO group at a dosage of 150 mg/kg. Tung et al. [22] reported that the flavonoids of *Acacia confusa* heartwood, (−)-2,3-*cis*-3,4-*cis*-3,3’,4,4’,7,8-hexahydroxyflavan, (−)-2,3-*cis*-3,4-*cis*-4’-methoxy- 3,3’,4,7,8-pentahydroxyflavan, melanoxetin, transilitin, and okanin, showed good inhibitory activity on xanthine oxidase activity, and significantly reduced the serum uric acid level by 66, 72, 75, 65 and 69 %, respectively, compared with the PO group. Zhu et al. [34] also found that administration of the flavonoids quercetin and rutin significantly reduced the serum uric acid level by 36 % and 32 %, respectively, at dosages of 150 mg/kg. Comparisons of the aforementioned results indicated that the major flavonoids from the *R. oldhamii* leaf extract have an excellent effect on reducing urate levels. Among the flavonoid constituents examined, hyposide, guaijaverin, and quercitrin were comparable with benzbromarone and showed an excellent effect on reducing urate levels.

## Conclusion

It is well-known that ROS have a high correlation with several diseases, such as ageing, atherosclerosis, inflammatory injury, cancer, and cardiovascular disease. This study demonstrated, for the first time, that among the leaf extracts of *R. oldhamii*, the EtOAc fraction exhibited the highest antioxidant activity. Thus, the EtOAc fraction was applied to the online HPLC–DPPH method, and six specific and excellent antioxidants were detected and identified. In addition, the study also demonstrated that the major constituents of *R. oldhamii* leaf extracts possessed potent in vivo hypouricemic effects in hyperuricemic mice pretreated with potassium oxonate. Thus, the dietary use of *R. oldhamii* leaf extracts and their constituents may provide some options for the prevention and/or treatment of hyperuricemia.
